# The First Report on the Medicinal Use of Fossils in Latin America

**DOI:** 10.1155/2012/691717

**Published:** 2011-09-29

**Authors:** Geraldo Jorge Barbosa Moura, Ulysses Paulino Albuquerque

**Affiliations:** ^1^Laboratory of Herpetology and Paleoherpetology Studies, Biology Department, Federal Rural University of Pernambuco, Rua Dom Manoel de Medeiros, s/n, Dois Irmãos, 52171-900 Recife, PE, Brazil; ^2^Laboratory of Applied Ethnobotany, Biology Department, Federal Rural University of Pernambuco, Rua Dom Manoel de Medeiros, s/n, Dois Irmãos, 52171-900 Recife, PE, Brazil

## Abstract

There have been very few ethnopharmacological studies performed on the traditional use of fossil species, although a few records have been conducted in Asia, Africa, and Europe. This study is the first ever to be performed on the use of Testudine (turtle) fossils for folk medicine in Latin America. An investigation was conducted in the Araripe Basin, which is one of the most important fossil-bearing reserves in the world due to the diversity, endemism, and quality of preservation of its fossils. We propose the formalization of a new discipline called ethnopaleontology, which will involve the study of the dynamic relationship between humans and fossils, from human perception to direct use.

## 1. Introduction

While most ethnopharmacological reports address the use of plants and animals in traditional medicinal practices [1–6], studies on the medicinal use of minerals are less common. The use of fossils for traditional remedies is an under-explored aspect of ethnopharmacology. Fossils are found in the myths and narratives of many different cultures and are used for traditional medicinal practices around the world [7, 8]. In a recent review of the worldwide use of fossils, Geer and Dermitzakis [7] reported on the use of belemnites, ammonites, and trilobite fossils, as well as echinoid, brachiopod, oyster shells, shark teeth, and mammal fossils, primarily in Africa, Asia, and Europe. Despite this report detailing evidence of fossil use, ethnoecological studies focusing on the relationship between human cultures and fossils are scarce. This relationship is complex and involves not only cultural and social aspects but also scientific and economic aspects because many scientific discoveries are a result of mining activity around the world. According to Mayor [9, 10], the relationship between humans and fossils is very ancient, and evidence of it can be found in the folk traditions of diverse people groups. Mayor reports that ancient Greeks and Romans “collected, measured, displayed, and pondered the extinct bones of beasts, and they recorded their discoveries and imaginative Interpretations of the fossil remains in numerous writings that survive today” [10].

This study focuses on the use of Testudine fossils for medicinal purposes in Latin America and is based on a casual record made during paleoherpetological investigations in the city of Nova Olinda, Ceará State, which is located in northeastern Brazil. Living reptiles stand out among vertebrates used for zootherapeutic purposes and are commonly used for traditional medicine on every continent [5, 6, 11–17]. They are also used for many other purposes: as pets, for food, for magicoreligious purposes, and for crafts [5, 6, 11, 16–24], which explains their significant presence in the animal trade [25] and the perceived need for conservation initiatives [26, 27].

Unlike living natural resources, fossil populations do not require management for their sustainability, but it is worth considering the importance of regulating their use by human societies [28]. They fit into the category of “World Heritage”, as they represent a door to the past and provide scientists with the opportunity to investigate the history of the planet and of life [28, 29].

 The study discussed in this paper was done in the Araripe Basin, known for being from the Cretaceous period, which is abundant, biologically diverse; and preserved well [30]. The Crato, Ipubi, and Romualdo Formations are important areas in the Araripe Basin [31], not only because of their fossil reserves of unparalleled scientific interest but also because they exist as a result of the ornamental rock trade, with over 372 mining companies exploiting the Cretaceous limestone from the region [32]. Mining activity has been responsible for the discovery of several fossil species that have contributed to our understanding of the evolution of paleobiodiversity; however, it has also resulted in increasing numbers of fossils being destroyed before the scientific community has gotten a chance to analyze them [33].

## 2. Material and Methods

**Figure 1:**
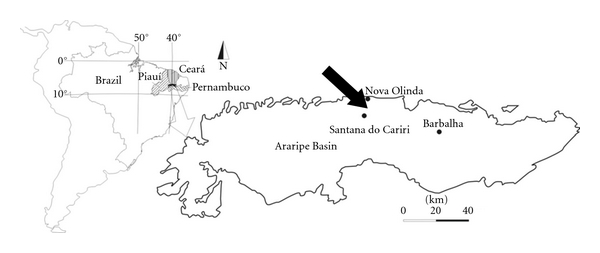
Location of the Caldas Quarry in the municipality of Nova Olinda, Ceará, northeastern Brazil.

The Araripe Basin (Figure 1) is a sedimentary unit located in the states of Pernambuco, Ceará, and Piauí, which are located inland in northeastern Brazil, between 38°30′ and 40°50′ W longitude and 7°05′ and 7°50′ S latitude. It covers a land area of 8,000 km^2^ [30].

The findings presented in this paper were casually recorded during field sampling performed in 2005 by G. J. B. Moura [30, 33–36] in the quarries of the Crato Formation. The study consisted of informal conversations with three workers from the Portland Cement Company working in the Caldas Quarry, which is in the municipality of Nova Olinda, Ceará, between the cities of Santana do Cariri and Nova Olinda, on the right side (from Nova Olinda to Santana do Cariri) of state Highway CE-255, which connects both cities. The Caldas Quarry is known for its significant mining activity in the region [32, 33] and as the discovery site of several new fossil species, including Anura [30, 33–35], Testudines [37], pterosaurs [38], and crocodilians [39]. The perceptions of quarry workers regarding the significance of fossil findings in this area were important considerations for this study. Although there are still no study reports that include the perceptions of quarry workers, the authors of this study are currently conducting research aimed at the development of such reports.

## 3. Results and Discussion

**Figure 2:**
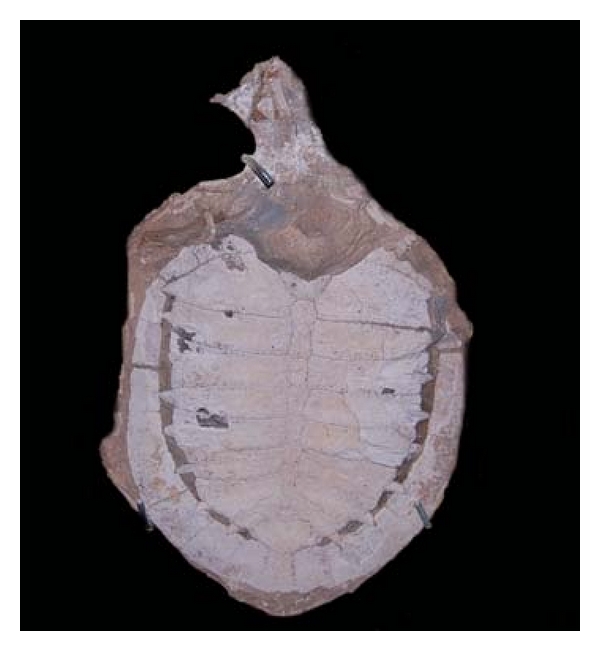
*Araripemys barretoi* Price, 1973 (Sauropsida-Testudine) deposited at the Museum of Santana do Cariri, Santana do Cariri-CE, reference number MPSC-V-010.

**Figure 3:**
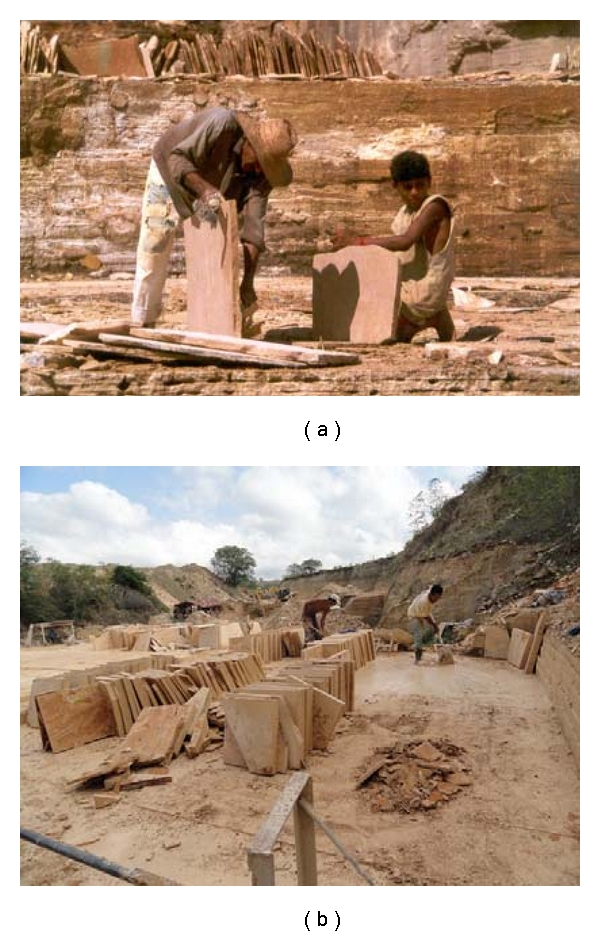
Workers at the Caldas Quarry cutting laminated limestone (photographs by Michel Fernandes Teixeira, 2009).

We collected information regarding the use of turtle shell fossils (Figure 2) as reported by quarry workers in the city of Nova Olinda (Figure 3). The shell is scraped and administered orally as a sedative, especially for very energetic, vigorous children. The turtle fossils originated from the Cretaceous strata of the Arrive Basin, especially from the Crato (Lacustrine Paleoenvironment), Ipubi (Lacustrine Paleoenvironment), and Romualdo (Marine Paleoenvironment) Formations, which belong to the Santana Group [31, 33].

Among the different species of Testudine fossils observed in the Araripe Basin, at least five species should be emphasized: (1) *Araripemys arturi* Fielding, Martill and Naish, 2005, belonging to the Pleurodira clade, (2) *Araripemys barretoi* Price, 1973, also belonging to the Pleurodira clade, (3) *Brasilemys josai* Lapparent de Broin, 2000, (4) *Cearachelys placidoi* Gaffney Campos and Hirayama, 2001 (Pleurodira), and (5) *Santanachelys gaffneyi* Hirayama, 1998 (Cryptodira). These species are mainly marine turtles [35, 40].

We propose the creation of a new discipline, ethnopaleontology, to study the dynamic relationship between humans and fossils, including aspects such as the cultural perception of fossils, fossil trade, and fossil use (mythical and direct). Ethnopaleontology differs from Medical Geology, which involves the study of the relationship between the geological environment and health issues of plants, animals, and people [41], and from Ethnopedology, which, according to Alves et al. [42] “consists of a set of interdisciplinary studies devoted to understanding the interfaces between the soil, the human species and other ecosystem components”. We argue that ethnopaleontology belongs within the scope of geomythology, which can be understood as “the science of recovering ancient folk traditions about complex natural process or extraordinary events [10]”. According to Mayor [9]:

“Native Americans observed, collected, and attempted to explain the remains of extinct invertebrate and vertebrate species long before contact with Europeans, and their cultural connection with fossils continues today. Their explanations, expressed in mythic language, were based on repeated, careful observations of geological evidence over generations”.

In ethnopaleontology, human attention is centered, not on the mineral composition of the fossil, but on the fossil itself as a representation of an organism that once lived, which includes the symbolic significance associated with such a representation. However, the most likely reason for the pharmacological use of fossils originates from the mineral elements that constitute them; this is the link between ethnopaleontology and medical geology.

Among the therapeutic uses for fossils, only those involving mammal fossils have been previously reported; they have been used as sedatives and for the treatment of several ailments, including diphtheria, sore throat, high stress, heart and liver problems, insomnia, manic behavior, excessive perspiration, night sweats, and chronic diarrhea (e.g., [7]). Testudine species are currently used in traditional medicine in Latin America to treat arthritis (*Gopherus flavomarginatus*, Leglier 1959), catarrh, erysipelas, bronchitis, asthma (*Chelonoidis carbonaria* Spix, 1824), sore throat, rheumatism, hernias, wounds, leishmaniasis, varicocele, earaches, female issues, asthma and pain (*Chelonoidis denticulata* Linnaeus, 1766) (e.g., [2, 5, 6]). They are also used to control thirst.

In northeastern Brazil, species that move slowly, such as *Uranoscodon superciliosus*, are typically used as a sedative; however, there is no record of the use of Chelonidade or Testudinidae as sedatives in Latin America (e.g., [2]). This type of use by imitative or mimetic association is common in folk medicine practices. The principles of sympathetic medicine are applied to cure afflictions that have a resemblance to the affected organ (e.g., [7]); however, the relationship as perceived by a culture may also involve mythological elements, as in the Afro-Brazilian cults in northeastern Brazil, where plants or animals are used for certain purposes according to the deity that “owns” that particular resource [4, 43].

The potential pharmacological activity of fossils may be scientifically explained by their mineral composition. Minerals have been used in medicinal practices of different cultures for different purposes, from topical (e.g., to treat skin ailments) to internal use [44]. For instance, Park et al. [44] observed antibacterial activity in a mixture of minerals containing sericite, talc, and halloysite. The association between medical geology and ethnopaleontology approaches can undoubtedly help us advance our knowledge of the pharmacological properties of minerals and the traditional medical systems that use such resources for health care purposes. This paper highlights the research possibilities in a region of great scientific importance, the Araripe Basin, and expands our understanding of the use of such resources in traditional medical systems.

Because of the substitution of living components with minerals during the lithification process, fossils may concentrate minerals of scientific and commercial interest and, therefore, like flora and fauna, are considered to be national property and are legally protected by Article 20 of the Brazilian Federal Constitution of 1988. We would like to underscore Decree no. 98830 of 1990, regulating the collection of fossils by foreigners; Law no. 8176 of 1991, establishing the exploitation of fossils without authorization from DNPM as a crime against the Union; and Law no. 9605 of 1998, establishing sanctions/penalties for crimes against fossiliferous property in the country. Although the collection of fossils is regulated in Brazil, most fossiliferous reserves are subject to illegal practices. The Araripe Basin, for example, has been the target of a countless number of illegal actions regarding fossils [28, 45]. Thus, it is important to develop more efficient conservation programs to preserve these rare traces of past life accumulated over geological time, which, in addition to enabling the understanding of biological evolution, may also hold materials of great interest to the socioeconomic development of the country.
